# Study of the intentional replantation procedure used to treat a tooth with a palatogingival groove: A case report

**DOI:** 10.4317/jced.59099

**Published:** 2022-02-01

**Authors:** Litao Yao, Jinsong Liu, Zheng Cao, Lieping Sheng

**Affiliations:** 1 Department of Dentistry, Sir Run Run Shaw Hospital, School of Medicine, Zhejiang University, Hangzhou 310058, Zhejiang, P.R. China; 2 Stomatology Hospital, School of Stomatology, Zhejiang University School of medicine, Clinical Research Center for Oral Disease of Zhejiang Province, Key Laboratory of Oral Biomedical Research of Zhejiang Province, Cancer center of Zhejiang University, Hangzhou 310006; 3 School and Hospital of Stomatology, Wenzhou Medical University, Wenzhou, 325027, China

## Abstract

In order to clarify the prognosis of intentional replantation used for palatogingival groove treatment for long-term follow-up observation, the case of a patient with a maxillary lateral incisor with palatogingival groove was investigated. The intentional replantation was carried out to preserve the tooth. The periodontal pocket and the apical bone defect were almost completely repaired at 12-month follow-up. However, the infection was reoccurred after 25-month follow-up examinations. The infected tooth was extracted, of which the root was investigated by histological analysis. Therefore, the reason of the replant failure and the pathways of bacterial infection was investigated.

** Key words:**Palatogingival groove, intentional tooth replantation, bacterial infection, maxillary lateral incisor.

## Introduction

A palatogingival groove is a developmental anomaly primarily involving the maxillary lateral incisors and usually begins near the cingulum of the tooth and extends along the root to varying lengths (1). The anomaly is sometimes associated with the onset of periodontal inflammation, which is likely to decrease the survival of the affected tooth. It is rare with a prevalence rate of 2.8% to 8.5% and occurs primarily on the lingual surface of the maxillary lateral incisors (2).

Multiple case reports have described various treatment methods that have been used to treat palatogingival groove ranging from periodontal regeneration to apexectomy, depending on the extent of the infection (3). The treatment goals of the palatogingival groove tooth include complete eradication of microorganisms, total and permanent sealing of the root groove that communicates between the root canal and the periodontium, and complete healing of the periodontium (4). To achieve effective treatment, clinicians first should determine whether the pulp is involved in the pathological changes identified during periodontal exploration. From an infection pathway point of view, the apical pulp might be infected via apical foramen when the infection occurs from the groove. Therefore, the endodontic treatment should be conducted if the pulp is infected.

The combination of nonsurgical endodontic treatment and periodontal regenerative surgery has been indicated effective (5). The favorable periodontal condition is essential for surgery. Therefore, intentional replantation combined with guided bone regeneration are considered as effective methods to preserve the tooth with insufficient periodontal support (6). In addition, the criteria for case selection remain controversial. Because teeth with extensive endodontic-periodontal lesions always present an unaccepTable periodontal status, it is uncertain whether intentional replantation is an appropriate treatment procedure for teeth with a palatogingival groove.

This report describes a patient with a maxillary lateral incisor with a palatogingival groove who was treated with modern intentional replantation. At one year after replantation, periapical and periodontal healing was observed. However, at 25 months, infection was represented, and the tooth was extracted finally. Thus, the extracted tooth with the palatogingival groove was decrowned. The resected root was placed in a fixative solution, and the histological analysis was completed to investigate the process and mechanism of the infection. The resected crown was used for the pontic of the temporary bridge restoration.

## Case Report

**Figure 1:**
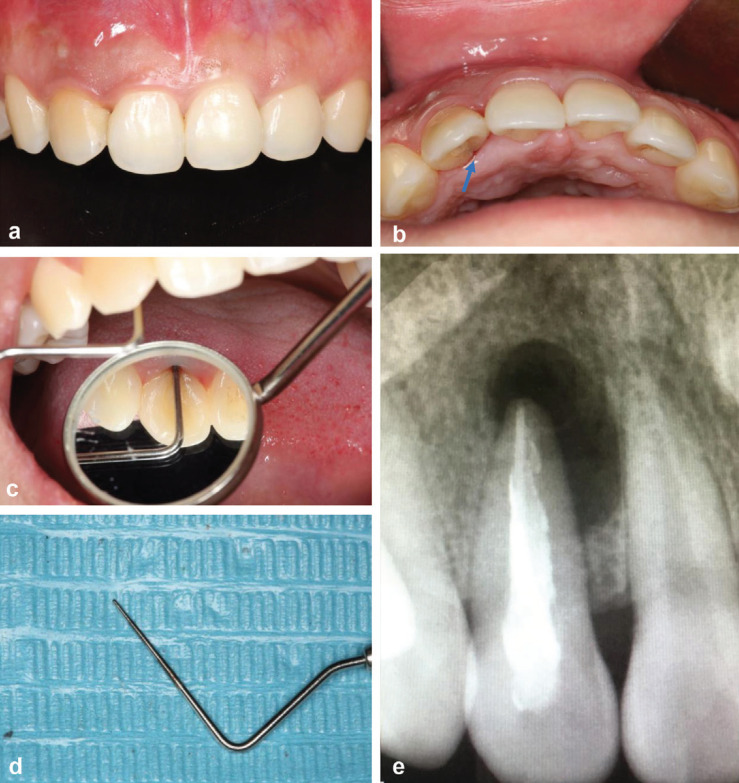
(a) The maxillary right lateral incisor. A preoperative clinical photograph shows a draining sinus tract on the labial gingiva. (b, c, d) A palate-gingival groove was detected on the maxillary right lateral incisor, accompanied by a 15-mm periodontal pocket in the mid palatal region. (e) the X-ray radiograph shows the root canal treated image and oval shadow.

A 25-year-old woman in good general health came to the dental clinic department. She complained of pus discharging from her gingiva and discomfort in her maxillary right anterior quadrant. It had continued for six months, even the tooth was treated at another hospital. Based on the initial clinical examination, it showed that the labial mucosa of the maxillary right lateral incisor was swelling and a draining sinus tract was on it and exhibited positive responses to percussion and palpation (Fig. 1a). The clinical examination revealed the palatal access was completely filled with light curing composite and a subtle groove that extended from the cervical to root on the palatal side (Fig. 1b). The examination also revealed a deep, narrow periodontal pocket (PD=15mm) in the mid-palatal region with probing bleeding (Fig. 1c,d). The maxillary right lateral incisor was in physiological level with no pain and abnormal occlusion can be observed. Radiographs confirmed the upper right lateral incisor was treated with root canal therapy. A slightly sparse filling in the apical segment with an extensive periradicular radiolucency (4*9 mm) was detected, which was associated with the affected tooth (Fig. 1e). The clinical and radiographic findings were consistent with the diagnosis of severe apical infection and a periodontal lesion associated with a palatogingival groove in the maxillary right lateral incisor.

**Figure 2:**
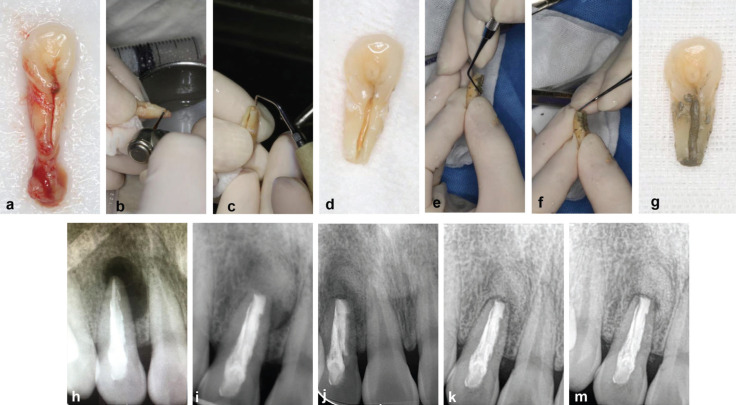
(a) The extracted maxillary right lateral incisor with apical granuloma was placed in a sterile gauze soaked with saline solution. (b) the manipulation of apexectomy. (c) the manipulation of retrograde preparation. (d) The palate-gingival groove was prepared with a fissure bur, and the apical 3 mm of the root apex was resected and retrogradely prepared. (e) the palate-gingival groove was filled with MTA. (f) the root apex was filled with MTA. (g) the tooth presented that both the palate-gingival groove and the root apex were filled with MTA. (h) Preoperative radiograph. (i) Radiograph taken after treatment (the apical region was filled with Bio-oss). (j) Radiograph taken at 2-month follow-up (k) Radiograph taken at 6-month follow-up (similar density with the surrounding bone tissue in the apical area and the obvious periodontal healing) (m)Radiograph taken at 12-month follow-up (similar density with the surrounding bone tissue in the apical area and the obvious periodontal healing).

An interdisciplinary treatment plan was formulated that included basic periodontal treatment, root canal retreatment under a microscope, intentional replantation, extraction, and implant surgery that utilized a bone graft after removing the affected tooth. All of the advantages and disadvantages of the treatment plan were provided to the patient who desired to keep this natural tooth. Moreover, at the request of the patient, the intentional replantation was conducted after the sign of informed consent. First, the maxillary right incisor was extracted with minimal force under local anesthesia, and extensive inflammatory granulation tissue was completely removed (Fig. 2a). Next, the tooth was held gently by the crown using physiological saline-soaked gauze (Fig. 2b). An apicoectomy was performed under an endodontic microscope (S100/OPMI Pico, Carl Zeiss Meditec, Jena, Germany), and the apical 3 mm of the root apex was resected (Fig. 2b). The palatogingival groove was ground with a fissure bur to remove the granulation in the groove. Subsequently, Ultrasonic irrigation (Satelec P5 Newtron, Acteon, Merignac, France) was used to complete the retrograde preparation (Fig. 2c), and the prepared tooth is seen in Fig. 2d. Both the palatogingival groove and the root apex were completely filled with Pro Root MTA (Dentsply Sirona, China) (Fig. 2e,f).

Bio-oss bone power (0.25g) (Geistlich, China) was placed into the apical bone defect area to enhance bone regeneration. And then, the tooth was replanted into its alveolar socket using a silicone rubber guide to confirm the accurate tooth placement. The entire procedure lasted 13 minutes. After replantation, the tooth surface was treated with acid etching and elastic fixation. At 12-month follow up, the X-ray examinations indicated the complete formation of new bone around the root apex, which showed the similar density with the surrounding bone tissue and the obvious periodontal healing (PD < 3mm). At the preoperative, postoperative, 2-, 6-, and 12-month follow-up examinations, periapical and periodontal healing was observed as time progressed (Fig. 2h-m). Furthermore, no deep narrow periodontal pocket (PD < 3mm) can be detected in the mid-palatal region at 12-month follow-up examinations. Occlusal adjustment was performed at each follow-up examination to avoid occlusal trauma to the maxillary right lateral incisor.

**Figure 3:**
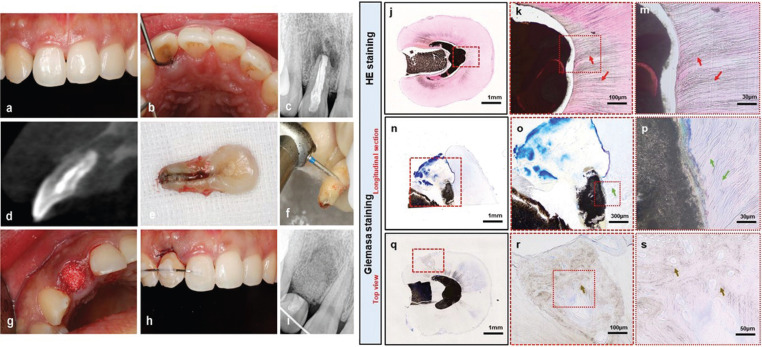
(a,b) Clinical photograph taken 25-month follow up. (a: the obvious pustule was reoccurred; b: a deep, narrow periodontal pocket in the mid-palatal region was reoccurred) (c) Radiograph taken 25-month follow up showed the reoccurred apical shadow. (d) the cone-beam CT evaluation of the upper right lateral incisor showed the obvious apical shadow and the resorption of palatal bone plate. (e) the photo of extracted tooth. (f) the manipulation of tooth cut off. (the root section was used for histological analysis and the crown section was used for temporary bridge restoration). (g) filling the extraction socket with bio-oss for the alveolar site preservation to provide enough bone for implantation in the future. (h) Temporary bridge restoration after the alveolar site preservation. (i) Radiograph taken after the temporary bridge restoration. Histological analysis of the extracted tooth root: (j), (k) and (m) HE staining of the cross section of the upper root. (the red arrows in b, c showed the obvious bacteria existed in the dentin tubules). (n), (o) and (p) Longitudinal section of the apical area of the root. (the green arrows in e, f showed the clear dentin tubules with no bacteria can be observed). (q), (r) and (s) Giemasa staining of the cross section. They showed the external root resorption at cervical area. (the brown arrows in h, i showed the osteocytes).

Unfortunately, at the 25-month follow-up examination, the patient reported experiencing discomfort with the treated tooth, and the pustule had reappeared (Fig. 3a). A deep narrow periodontal pock*et al*so reoccurred at the palatal surface of the tooth (Fig. 3b). Moreover, radiograph and the cone-beam CT evaluation revealed a prominent resorption shadow at the palatal surface and apex of the root, which indicated an infection was present around the tooth root (Fig. 3c,d). Although, the tooth mobility was still in physiological level, no other effective method could be used to protect the tooth. Therefore, the tooth extraction was inevitable. Afterwards, the alveolar site preservation was performed for further implantation (Fig. 3g).

The extracted tooth was seen in (Fig. 3e). According to the periodontal and apical situation, the tooth will not be saved. The crown of the tooth was cut off using a high-speed turbine to allow for temporary bridge restoration (Fig. 3f) after the extraction. The socket was full filled with the bio-oss bone power (0.25g) (Geistlich) (Fig. 3g), of which the procedure and the final effect was displayed in Fig. 3h. The resected root was fixed in 4% paraformaldehyde for histological analysis.

The H&E staining of the top view of the cervical part of the tooth revealed an area that had been extensively invaded by bacteria (Fig. 3k and m, indicated by the red arrow), and the apical sealing area, when stained with Giemsa, showed clear dentin tubules with no discernible bacteria (Fig. 3o,p, indicated by the green arrow). In addition, the top view of the cervical part of the tooth showed that an extra root resorption area (Fig. 3q, indicated by the red frame) when stained with Giemsa, and showed the bone tissue structure and the obvious osteocytes in the root tissue (Fig. 3r,s, indicated by the brown arrow).

## Discussion

Intentional replantation is considered a last resort procedure prescribed only for “hopeless teeth,” as proposed by Grossman (7). However, the failure of the intentional replantation of teeth with a palatogingival groove often occurs. Numerous reasons have been cited for failure, including inadequate sealing of the palatal sulcus, infection and an extended time taken for manipulation. The palatogingival groove acts as a lesion for plaque formation and typically promotes the development of a combination of endodontic and periodontic disease. This disease progression can lead to a misdiagnosis of a palatogingival groove as a primary endodontic lesion. The successful treatment of a tooth with a palatogingival groove relies on effective endodontic therapy and the resolution of the associated localized periodontal defect (8).

Moreover, the bacterial biofilm covered the entire apical area and the depression on the surface of the root. Thus, it is clear that relying on root canal retreatment using microscopy or periodontal treatment alone cannot ensure a satisfactory prognosis. All necessary procedures should be performed routinely to control and remove the infection irritants and protect the periodontal ligament. Therefore, additional essential details of the procedure were also followed, including using minimally invasive tooth extraction, keeping the tooth surface moist, performing the standard apical treatment, and strictly restricting the time that the tooth was out of the socket. Especially, the extraoral duration should be 15min or less, which plays a pivotal role in the prognosis of intentional replantation. Mainkar analyzed 713 cases in 2017 and found that the retention of intentional replantation reached 89.1%, and the occurrence of extra-root resorption was 3% to 4.9% (9). Therefore, as soon as possible, aided by the use of Hank’s balanced solution, rapid replantation can prevent the occurrence of complications such as extra-root resorption. Consequently, the treatment outcomes achieved in this case included no pockets that were deeper than 3mm, and resolution of the radiographic radiolucency at the 12-month follow-up examination.

Several different materials have been used to seal palatogingival grooves (10). Glass ionomer cement is suitable because of its sealing properties and antibacterial action, but its solubility limits its applicability (11). Several resin-based materials have been introduced recently, but the long-term effects of their use need to be demonstrated with additional clinical cases (12). Compared with other materials, the MTA is regarded as a conventional material with excellent sealing property, mechanical ability, cytocompatibility and widely applied in previous cases (13,14). In addition, iRoot SP also showed effective sealing ability and biocompatibility, while MTA might provide more inductive potential and hard tissue deposition compared with it (15). Moreover, the using of grey or white MTA in intentional replantation was remained consideration. Gray MTA has a longer setting duration than white MTA, and its initial compressive strength is low (16).

The recent long-term follow-up research on intentional replantation has not been sufficient to investigate the failure of replantation of teeth with palatogingival grooves. From our perspective, the mechanism of bacterial infection is mainly attributed to the insufficient sealing which caused the microleakage and bacteria adhesion. Therefore, histological analysis of the extracted root, obtained at the 25-month follow-up, was conducted to confirm our speculation. The results indicated that the micro-leakage mainly occurred from the cervical area of the root canal. On the one hand, it was noted that the MTA used to seal the cervical area of tooth could be easily washed off, which severely compromised the sealing effect (17). On the other hand, the anatomy of the tooth neck and the upper part of the root canal were in close proximity to the external environment. bacteria and gingival crevicular fluid could have easily permeated the sealed region and caused an inflammatory reaction. Therefore, the effective method to seal the neck and upper end of the root canal and provide superior biological property are critical for the long-term preservation of the replanted tooth with palatogingival grooves.

## Conclusions

The case we investigated in this study indicated that the intentional replantation was successful within 12-months of follow-up; however, periodontal and apical infection reoccurred after 25-months of follow-up. The histological analysis of the tooth suggested that the source of the bacteria infection might have originated from the micro-leakage caused by insufficient groove sealing. In general, this problem requires further assessment *in vitro* and analysis of the underlying mechanisms, including a genetic study.
